# Monitoring Cannabinoid CB2 -Receptor Mediated cAMP Dynamics by FRET-Based Live Cell Imaging

**DOI:** 10.3390/ijms21217880

**Published:** 2020-10-23

**Authors:** Leonore Mensching, Sebastian Rading, Viacheslav Nikolaev, Meliha Karsak

**Affiliations:** 1 Center for Molecular Neurobiology (ZMNH), University Medical Center Hamburg-Eppendorf (UKE), 20246 Hamburg, Germany; l.mensching@uke.de (L.M.); s.rading@uke.de (S.R.); 2 Institute of Experimental Cardiovascular Research, University Medical Center Hamburg-Eppendorf, Martinistr. 52, D-20246 Hamburg, Germany; v.nikolaev@uke.de; 3 DZHK (German Center for Cardiovascular Research), partner site Hamburg/Kiel/Lübeck, Martinistr. 52, D-20246 Hamburg, Germany

**Keywords:** G-protein coupled receptor, cannabinoid, CB2, FRET, biosensor, cAMP, Epac1-camp, β-caryophyllene, live-cell imaging, signaling

## Abstract

G-protein coupled cannabinoid CB2 receptor signaling and function is primarily mediated by its inhibitory effect on adenylate cyclase. The visualization and monitoring of agonist dependent dynamic 3′,5′-cyclic adenosine monophosphate (cAMP) signaling at the single cell level is still missing for CB2 receptors. This paper presents an application of a live cell imaging while using a Förster resonance energy transfer (FRET)-based biosensor, Epac1-camps, for quantification of cAMP. We established HEK293 cells stably co-expressing human CB2 and Epac1-camps and quantified cAMP responses upon Forskolin pre-stimulation, followed by treatment with the CB2 ligands JWH-133, HU308, β-caryophyllene, or 2-arachidonoylglycerol. We could identify cells showing either an agonist dependent CB2-response as expected, cells displaying no response, and cells with constitutive receptor activity. In Epac1-CB2-HEK293 responder cells, the terpenoid β-caryophyllene significantly modified the cAMP response through CB2. For all of the tested ligands, a relatively high proportion of cells with constitutively active CB2 receptors was identified. Our method enabled the visualization of intracellular dynamic cAMP responses to the stimuli at single cell level, providing insights into the nature of heterologous CB2 expression systems that contributes to the understanding of Gαi-mediated G-Protein coupled receptor (GPCR) signaling in living cells and opens up possibilities for future investigations of endogenous CB2 responses.

## 1. Introduction

The endocannabinoid system consists of the G-protein coupled cannabinoid receptors CB1 and CB2, the main ligands that are derivatives of arachidonic acid and biosynthesizing and degrading enzymes. Expression and signaling of CB2 receptors have been associated in numerous pathophysiological conditions, such as in cardiac, neurodegenerative, hepatic, and cancer diseases [1,2,3,4].

First described in the early 1990’s by Munro et al. (1993) [5], the CB2 receptor has since developed into a promising target of biomedical research. Cannabinoid receptor ligands have been repeatedly associated with the treatment of cancer and anti-tumor activity [6]. Previous studies demonstrated an important and promising role of cannabinoids in cancer treatment being observed in different cancer cell lines and in breast, liver, and prostate cancer, as well as in glioblastoma and lymphoma [7].

As a Gαi/o -coupled GPCR, the activation of CB2 leads to the inhibition of adenylate cyclases (AC) via Gαi subunits [5,8], causing a decrease in 3’,5’-cyclic adenosine monophosphate (cAMP). Recently, CB2 signaling via Gαs has been demonstrated in human PBMCs [9] and binding to Gαs protein has been detected [10]. Early reports on CB2-mediated signaling from Bouaboula et al. [8] already registered a high degree of constitutive receptor activity in cAMP measurements in heterologous expression systems that was later confirmed by describing the action of CB2 inverse agonists [11,12]. To this date, it is not clear whether CB2 also exhibits constitutive activity in vivo and, if so, what the physiological role of this property might be.

The engagement of CB2 in specific cellular signaling pathways that are important to physiological and pathophysiological processes can be modulated by different CB2 ligands. Although there are many studies describing functional effects of CB2 ligand treatment in animal models and in vitro, only recently, research has targeted the functional selectivity of known and novel CB2 ligands to shed light on the mechanisms that elicit these effects [13].

The two most important and best described endogenous ligands that bind CB2, as well as CB1, are the endocannabinoids anandamide (AEA) [14] and 2-arachidonoylglycerol (2-AG) [15]. Both endocannabinoids are lipids that are generated from membrane phospholipids and they are Ca^2+^ -dependently released in response to stimuli. AEA and 2-AG both show a bias towards GIRK activation over cAMP signaling, whereas AEA is also biased towards the activation of MAPK ERK1/2 [13].

Phytocannabinoids are natural cannabinoid receptor ligands that are produced in plants. Trans-∆9-tetrahydrocannabinol (THC) is the most prominent phytocannabinoid, which is predominantly responsible for the psychoactive effect of recreational cannabis use mediated by CB1 [16]. Around 100 different phytocannabinoids have been found in plants of the genus Cannabis, among those cannabidiol. The terpenoid, β-caryophyllene (BCP), found in e.g., the cannabis plant, hops, rosemary, and basil has been reported to selectively bind human CB2 [17] and show CB2-mediated neuroprotective effects in mouse models, such as EAE [18], although subsequent studies have questioned this [19,20]. Synthetic cannabinoids that act as CB2 antagonists and inverse agonists have also contributed to identifying CB2 function. SR144528 and AM630 are inverse agonists that show the highest CB2 selectivity [13] and they have been used in various in vitro and in vivo studies. For cAMP signaling, both inverse agonists are highly potent and functionally selective over GIRK and ERK1/2 pathways [13,21]. In a study from Bolognini et al. [12], it was also shown that AM630 acts as a protean ligand at the human CB2 receptor.

It must be stressed that, to date, all CB2 ligand bias studies [13,21] have been performed in cell models and with receptor overexpression. Additionally, ligand bias has been determined relative to the nonselective, highly affine, and potent cannabinoid receptor agonist CP55,940, which, in turn, also shows a bias towards cAMP signaling [13,21]. Further limitation of former studies is the use of multicellular measurements that mainly determined the endpoint cAMP levels.

Over the last few years, advancements in the measurement of intracellular cAMP levels have significantly contributed to the understanding of the spatial and temporal control of cAMP-dependent signaling in cells. Transfectable biosensors that report cellular cAMP concentrations by bioluminescence resonance energy transfer (BRET) has been utilized to elucidate the temporal nature of agonists and allosteric modulators of the cannabinoid receptor CB1 [22] and CB2 [19,23].

The use of Förster resonance energy transfer (FRET)- based cAMP biosensors allows for the live measurement of cAMP levels in the whole cell or at particular subdomains on a single cell level [24,25]. Common for FRET-based cAMP sensors is that the binding of cAMP initiates a conformational change of the sensor that leads to a change in FRET between the two sensor-coupled fluorophores. The present paper examined the use of the cAMP biosensor Epac1-camps that were developed by Nikolaev et al. [26] for investigations on CB2 receptor related cAMP signaling. The sensor consists of the cAMP-binding domain of Epac1 and it is flanked by enhanced cyan fluorescent protein (CFP) and yellow fluorescent protein (YFP). The binding of cAMP leads to an increased distance between CFP and YFP and, therefore, to reduced FRET efficiency. Because of the design of the sensor, CFP and YFP are in equimolar concentration and the single cAMP binding domain allows for a rapid activation of the sensor when compared to, e.g., PKA-based sensors [27]. With an EC50 of 2.35 μM cAMP, Epac1-camps is suitable to sensitively detect cAMP in a physiological range of 0.1 μM to 10 μM [26,28].

To the best of our knowledge, this is the first description of investigating single cell characteristics of cannabinoids targeting the CB2 receptor. Through our approach of cAMP detection on a single cell live-imaging basis, we proved the reliability of the method by examining different ligands and we were able to determine ligand kinetics and maximal responses on cAMP. 

## 2. Results

### 2.1. Stable Expression of FLAG-hCB2 in Epac1-HEK Cells

**Figure 1:**
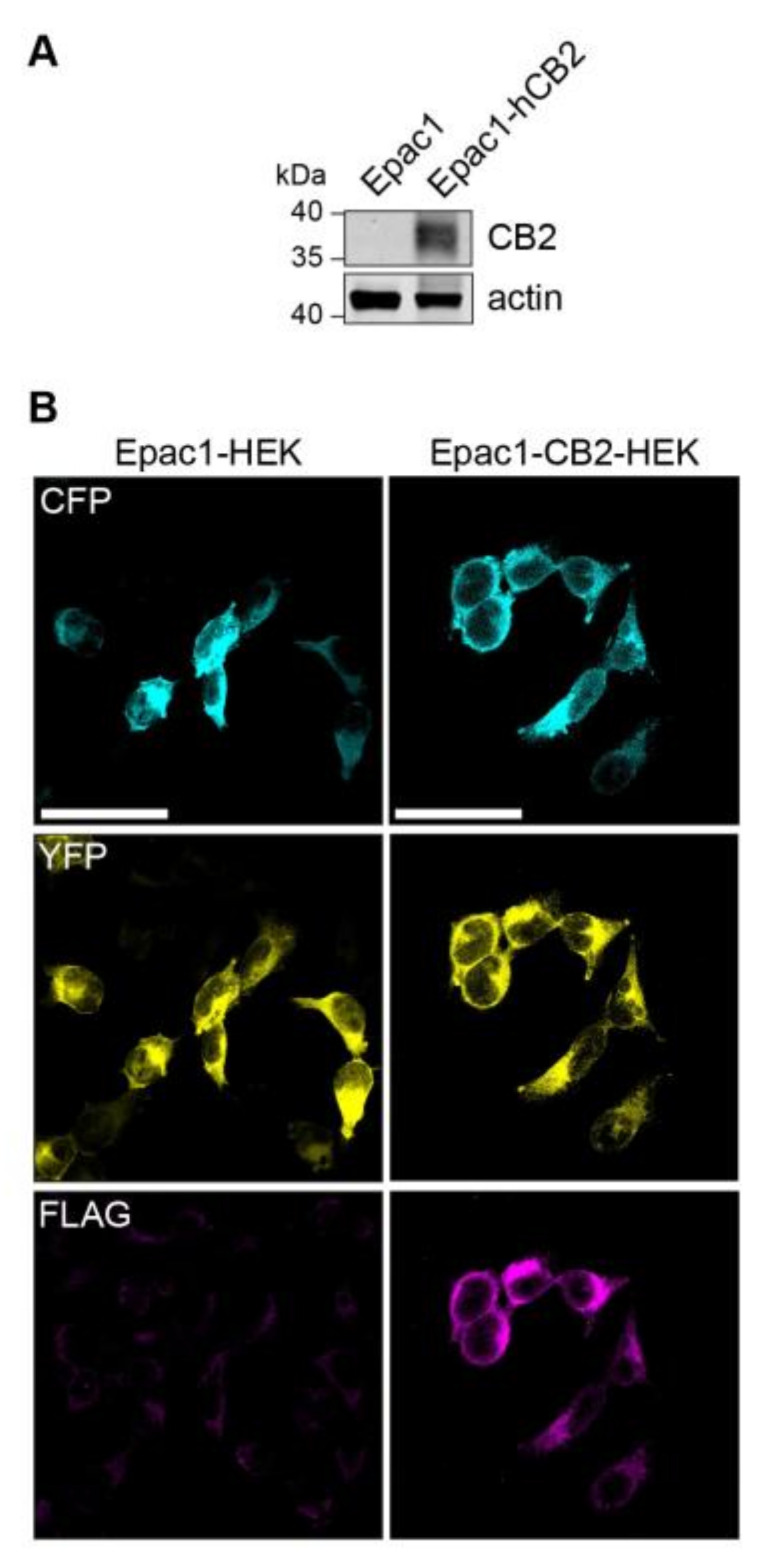
Expression of FLAG-CB2 and Epac1-camps in Epac1 and Epac1-CB2-HEK cells. (**A**) Western Blot of FLAG-CB2 and mock-transfected Epac1-HEK cells after selection process used in all further experiments with detection for CB2 receptor and actin as loading control. (**B**) Representative confocal microscopy images of immunofluorescent detection of Epac1-camps and FLAG-CB2. First column of images shows Epac1-HEK and the second column shows Epac1-CB2-HEK cells. Cyan fluorescent protein (CFP) (cyan) and yellow fluorescent protein (YFP) (yellow) fluorescent signals are seen in the first two rows respectively. FLAG-staining for FLAG-CB2 is seen in the third row of images (magenta). Scale bar = 50 µm.

A stable cell line expressing CB2 receptor was generated in order to generate a HEK293 cell model that allows for the live recording of CB2-mediated changes in intracellular cAMP levels. Therefore, a FLAG-tagged human CB2 (p.63 Q–p.316 H variant) protein encoding plasmid was transfected into Epac1-camps-HEK293 (short Epac1-HEK) that stably express the FRET-based cAMP-biosensor Epac1-camps. A selected CB2 expressing colony was propagated further and compared to Epac1-HEK cells for CB2 expression via immunoblotting, which showed no CB2 expression (Figure 1A). The immunocytochemistry of Epac1-CB2-HEK cells revealed the expression of Epac1-camps and FLAG-hCB2 (Figure 1B) using an antibody against the FLAG-tag. In Epac1-HEK cells, no fluorescence for FLAG-hCB2 was detected and homogenous cytosolic expression of Epac1-camps comparable to Epac1-CB2-HEK cells was seen.

### 2.2. Evaluation of Live Cell FRET Imaging and cAMP Measurement in Epac1-HEK Cells

**Figure 2:**
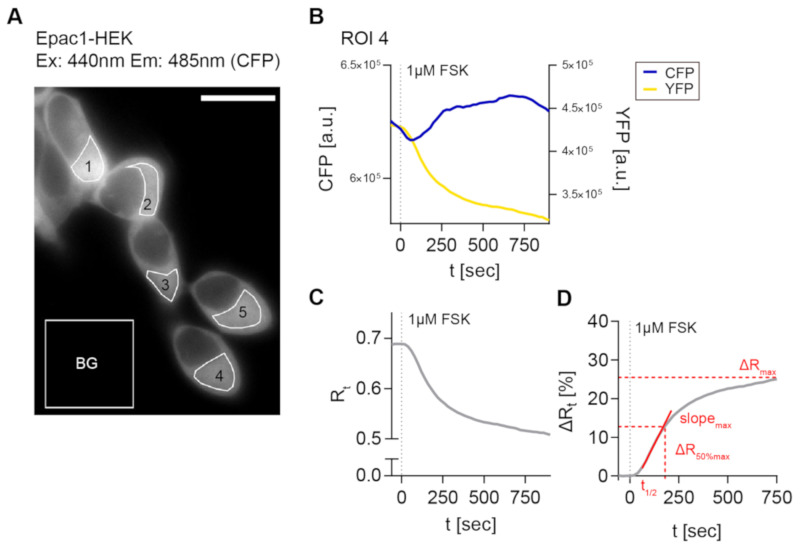
3′,5′-cyclic adenosine monophosphate-Förster resonance energy transfer (cAMP-FRET) imaging of Epac1-HEK cells and FRET data analysis. (**A**) Representative fluorescent CFP image of Epac1-HEK cells excited with 440 nm light, measured regions of interest (ROIs) and background ROI. (**B**) Individual bleedthrough-corrected time-dependent fluorescence intensity traces (blue CFP, yellow YFP) from ROI 4 in (**A**) after stimulation with 1 µM Forskolin (FSK). (**C**) Time-dependent FRET ratio Rt (grey) calculated from fluorescence intensities in (**B**). (**D**) Normalized, time-dependent FRET ratio changes ∆Rt calculated from (**C**) and analyzed FRET response parameters (red). Scale bar = 20 µm.

Next, the generated cell lines were used to establish a reliable FRET imaging and stimulation protocol in order to evaluate CB2-mediated cAMP signaling in real-time on single cell level. For the detection of Gαi -mediated inhibition of AC activity and cAMP production in model systems, it is often necessary to pre-activate the AC before applying a potentially inhibitory stimulus to elevate low cellular cAMP levels [29,30]. In a majority of cAMP studies, the direct AC activator FSK is used for this purpose [30]. Therefore, we used a 1 µM FSK solution and recorded different regions of interest (ROI) for their time dependent fluorescence signals for CFP and YFP and time-dependent FRET ratio (R) (Figure 2A–C). Figure 2D provides an overview of the FRET imaging method and analysis pipeline with analyzed response parameters.

**Figure 3:**
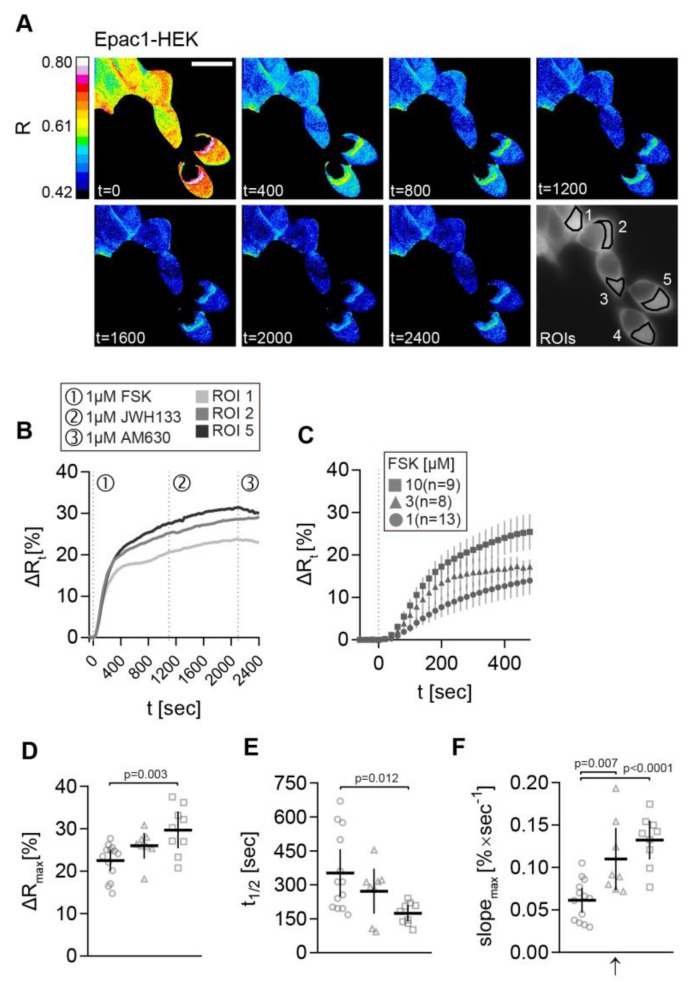
cAMP-FRET imaging of Epac1-HEK cells after stimulation with different concentrations of FSK. (**A**) Representative FRET ratio (R) images at different time points (t in seconds) and corresponding single cell FRET ratio (∆Rt) (**B**) traces from indicated Epac1-HEK cells stimulated with 1 µM FSK (1), 1 µM CB2 agonist HU308 (2) and 1 µM AM630 (3). (**C**) Mean ± 95%CI ∆Rt FRET ratio traces showing the first 480 s after stimulation of Epac1-HEK cells with 1 µM, 3 µM and 10 µM FSK. (**D**) Maximum peak ∆R, (**E**) t1/2, and (**F**) max. slope values (arrow represents slope direction) of FRET responses to varying concentrations of FSK in Epac1-HEK cells. Data were analyzed using Nested One-Way ANOVA with Tukey-adjusted p-values. Each independent data point n represents the average of all cells in one recording/coverslip. 1 µM FSK *n* =13, 3 µM FSK *n* = 8, 10 µM FSK *n* = 9. Mean ± 95%CI. Scale bar = 20 µm.

We stimulated the cells with increasing FSK concentrations to determine the optimal FSK concentration (Figure 3). Representative time-dependent FRET ratio images from a recording of Epac1-HEK cells stimulated with 1 µM FSK, followed by 1 µM CB2 agonist JWH133, and finally 1 µM CB2 inverse agonist AM630 is presented in Figure 3.

The corresponding ∆Rt line plots from selected ROIs (1, 2, and 5) show stimulation time points and the increase of ∆R after FSK stimulation in all ROIs (Figure 3B). No FRET change was observed after the application of CB2 ligands in Epac1-HEK cells. The average ∆Rt time traces of Epac1-HEK cells stimulated with 1 µM, 3 µM, and 10 µM FSK show the step-wise concentration-dependent increase in cAMP production within the first 480 s after FSK stimulation (Figure 3C). Maximal ∆R amplitudes, time to half maximum t1/2, and the maximal slope of the FSK responses (in positive direction) corroborate this observation.

When comparing the data from 1 µM, 3 µM, and 10 µM FSK recordings, larger ∆R amplitudes, shorter half-times, as well as steeper max. slopes of the signals were seen with increasing FSK concentration (Figure 3D–F). This was the most evident regarding the 10-fold concentration increase from 1 µM to 10 µM FSK. The max. slope of the FRET response showed significant differences between all of the concentration steps (Figure 3F), leading to the possible conclusion that this parameter most accurately represents the changes in cAMP accumulation caused by FSK (1 µM FSK vs. 3 µM FSK max. slope: M = −0.0465, 95% CI = −0.0813, −0.0117, *p* = 0.0073; 1 µM FSK *vs*. 10 µM FSK max. slope: M = −0.0708, 95% CI = −0.1048, −0.0369, *p* < 0.0001).

### 2.3. FRET Recordings from FSK-Stimulated Epac1-HEK Cells Showed the Feasibility of the Image Acquisition and Analysis Pipeline

For investigation of CB2 signaling in Epac1-CB2-HEK cells, a concentration of 1 µM FSK was chosen as the initial pre-stimulation of ACs. The stimulation of Epac1-HEK cells with 1 µM FSK showed that the response parameters are suitable for a subsequent stimulation with CB2 agonists (∆Rmax: M = 22.49, 95% CI = 20.00, 24.99; t1/2: M = 352.1, 95% CI = 247.5, 456.8; slope: M = 0.0615, 95% CI = 0.0469, 0.0761; *n* = 13) and the response was not significantly slower than the FRET response to 3 µM FSK. Although the FRET responses to 10 µM FSK were, on average, faster and larger (∆Rmax: M = 29.72, 95% CI = 25.37, 34.06; t1/2: M = 174.3, 95% CI = 137.8, 210.9; slope: M = 0.1324, 95% CI = 0.1097, 0.1552; *n* = 9), choosing 1 µM FSK minimizes the risk of masking the anticipated Gαi -mediated inhibition of cAMP production after CB2 activation.

### 2.4. Live Measurement of CB2-Mediated cAMP Dynamics Revealed Different CB2-Mediated cAMP Response Patterns in Epac1-CB2-HEK Cells

Next, we aimed to establish a protocol for single cell live recordings of CB2-mediated cAMP signaling. To this end, the cells were stimulated with 1 µM FSK to sub-maximally activate ACs and elicit cAMP production. After a baseline was reached, the cells were stimulated with different CB2 agonists to activate CB2 and inhibit cAMP production via Gαi subunits. Epac1-CB2-HEK cells were then stimulated with 1 µM AM630, a CB2-selective inverse agonist, in order to block recorded responses to CB2 agonists and show their CB2 specificity.

**Figure 4:**
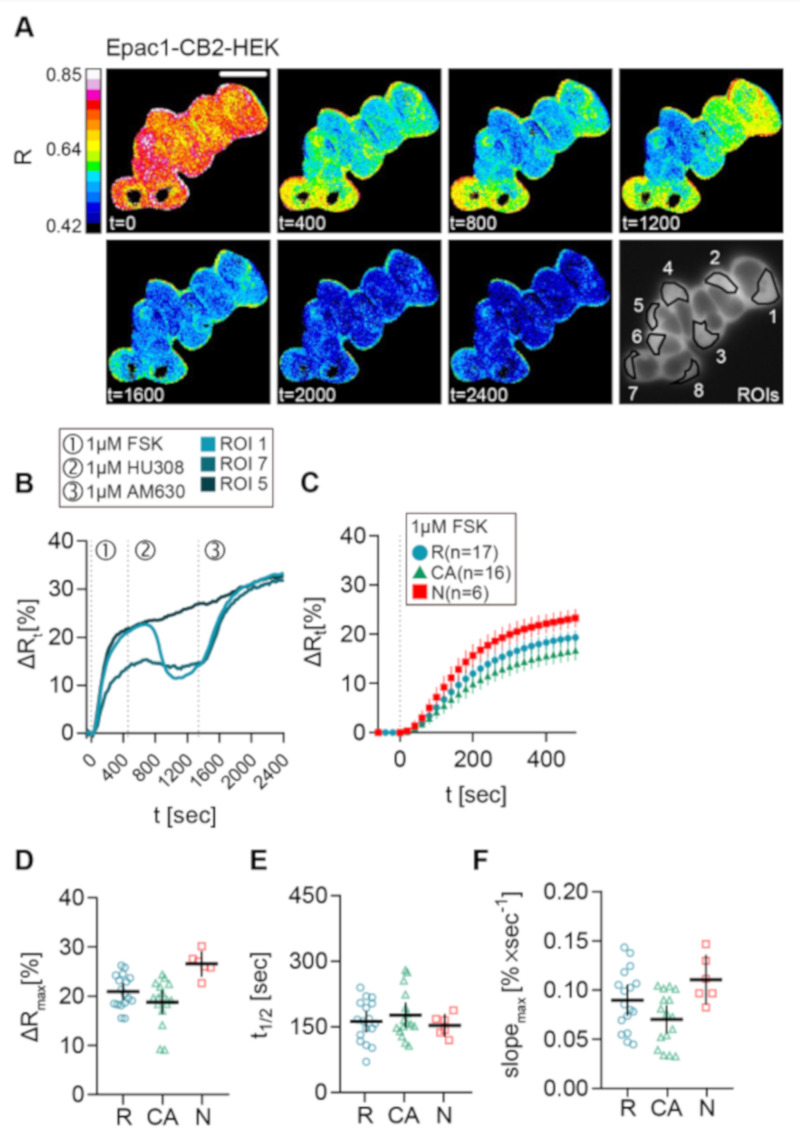
FSK response of different CB2 response types in Epac1-CB2-HEK cells. (**A**) Representative FRET ratio (R) images at different time points (t in seconds) and corresponding single cell FRET ratio (∆Rt) (**B**) traces from indicated cells stimulated with 1 µM FSK (1), 1 µM CB2 agonist HU308 (2) and 1 µM AM630 (3) showing the three response types R (ROI 1), CA (ROI 7), and N (ROI 5). (**C**) Mean ± 95%CI ∆Rt FRET ratio traces showing the first 480 sec after FSK stimulation of the different Epac1-CB2-HEK response types. (**D**) Maximum peak ∆R, (**E**) t1/2 and (**F**) max. slope values of FRET responses to FSK (arrow represents slope direction) of the different Epac1-CB2-HEK response types. Each data point n in D, E, and, F represents the average of all cells in one recording/coverslip with at least three cells of a response type. Type R *n* = 17, Type CA *n* = 16, Type N *n* =6. Mean ± 95%CI. Scale bar = 20 µm.

Representative live-cell recording of a group of Epac1-CB2-HEK cells stimulated with FSK, followed by HU-308, and finally by AM630 addition are presented as the processed FRET ratio (R) images in Figure 4A. These cells showed differences in their cAMP response to the stimulation protocol.

Unexpectedly, the data that were acquired from this stimulation scheme revealed three response types (Figure 4), which all showed a clear relative increase of ∆R and, therefore, intracellular cAMP levels after stimulation with FSK: (1) ROI 1 as type R (responder), a “classical” cellular response with a detectable inhibition of FSK-mediated cAMP production after CB2 agonist stimulation, that was blocked by AM630; (2) ROI 7 as type CA (constitutive activity), no response of the cell to a CB2 agonist, but a detectable increase in cAMP following stimulation with AM630 comparable to the reported effects when a GPCR shows a high degree of constitutive activity [12]; (3) ROI 5 as type N (non-responder), no response to either CB2 ligand. Representative results with stimulation time points and individual cellular responses given in the corresponding ∆R line plots of three chosen cells display the three different response types that were observed in Epac1-CB2-HEK cells (Figure 4B). The experiments were performed for the synthetic CB2 agonists JWH-133 and HU-308 and for the natural cannabinoids BCP and 2-AG.

Next, we investigated the observed response patterns and then characterized them in detail. Therefore, we pooled all of the FSK-response parameters from all recordings of Epac1-CB2-HEK cells with the different CB2 agonists and analyzed them according to response type. Only small differences in the FRET response were seen (Figure 4C–F). Within the first 480 s after FSK application, cells with response type N showed the largest FRET change, which is depicted in Figure 4C in averaged ∆R line plots. The maximal ∆R (M = 26.58, 95% CI = 24, 29.15) as well as max. slope values (M = 0.1106, 95% CI = 0.0855, 0.1356) (*n* = 6) were also increased in type N responders as compared to R or CA types. On average, type R responses were only marginally larger (M = 20.94, 95% CI = 19.21, 22.68) and steeper (M = 0.0898, 95% CI = 0.074, 0.1056) (*n* = 17) than the responses from type CA cells (Figure 4C,D,F). Because, sometimes, three or more ROIs of two different response types were seen in one recording, the data are partially paired, and further statistical test were not applied. Average half time values from the different response types that were around 150 s did not differ (Figure 4E).

**Figure 5:**
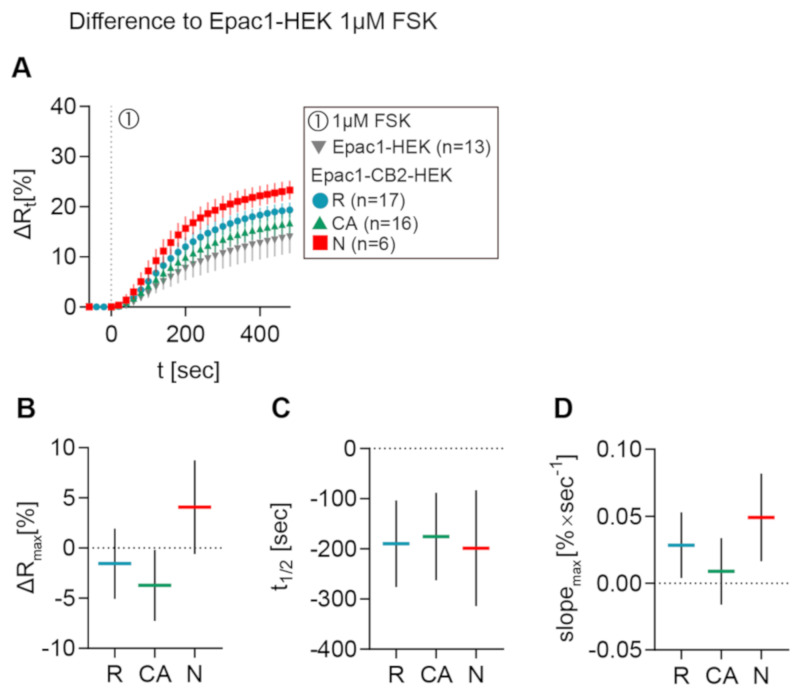
Differences in FRET response parameters after FSK stimulation between Epac1-CB2-HEK response types and Epac1-HEK cells. (**A**) Mean ± 95%CI ∆Rt FRET ratio traces showing the first 480 s after FSK stimulation of the different Epac1-CB2-HEK response types compared to Epac1-HEK cells (taken from 6.3C and 6.4C). (**B**) ∆R, (**C**) t1/2, and (**D**) max. slope mean ± 95%CI differences between Epac1-CB2-HEK type R, CA, and N responders to Epac1-HEK cells that were stimulated with 1 µM FSK. Mean ± 95%CI.

In Figure 5A, the mean ± 95% CI ∆Rt FRET ratio traces of type R, CA, and N Epac1CB2-HEK cells are depicted together with Epac1-HEK cells after 1 µM FSK stimulation. Figure 5B–D shows the mean ± 95%CI differences between response parameters from type R, CA, and N cells to the results of the Epac1-HEK cell stimulation with 1 µM FSK. Max. ∆R values of type R cells stimulated with 1 µM FSK differed the least when compared to control cells (M = −1.55, 95%CI = −5.04, 1.94), but they showed lower max. ∆R on average. Type CA showed max. ∆R responses smaller than the control cells with a larger difference than type R (M = −3.72, 95%CI = −7.25, −0.19). A higher FSK-elicited max. ∆R as compared to Epac1-HEK cells was seen for Epac-CB2 type N cells (M = 4.08, 95%CI = −0.59, 8.75) (Figure 5B).

FRET responses from Epac1-CB2-HEK cells to FSK had faster half-time values when compared to control cells in all response types (R: M = −190, 95%CI = −276, −104; CA: M = −175, 95%CI = −263, −88; N: M = −198, 95%CI = −314, −83) (Figure 5C). Max. slopes of FRET responses from type CA showed the smallest difference to the control cells (M = 0.0089, 95%CI = −0.0159, 0.034), whereas the type R and N max. slopes were steeper by a higher margin (R: M = 0.0283, 95%CI = 0.0039, 0.0528; N: M = 0.0491, 95%CI = 0.0164, 0.0819) (Figure 5D). The analysis of FSK-elicited FRET responses in Epac1-CB2-HEK cells showed that, through functional CB2 receptors, either possibly constitutively active (type CA) or not (type R), the cAMP production upon direct AC activation via FSK might already be affected in magnitude and speed. The presence of different GPCR conformations and their abundance at the membrane has been shown to influence the availability of Gαi and Gβγ subunits as well as AC activation via FSK or Gαs subunits specifically in heterologous expression systems [11,12].

### 2.5. Reduction of cAMP Levels After CB2 Activation with Different Agonists in Epac1-CB2-HEK Cells

**Figure 6:**
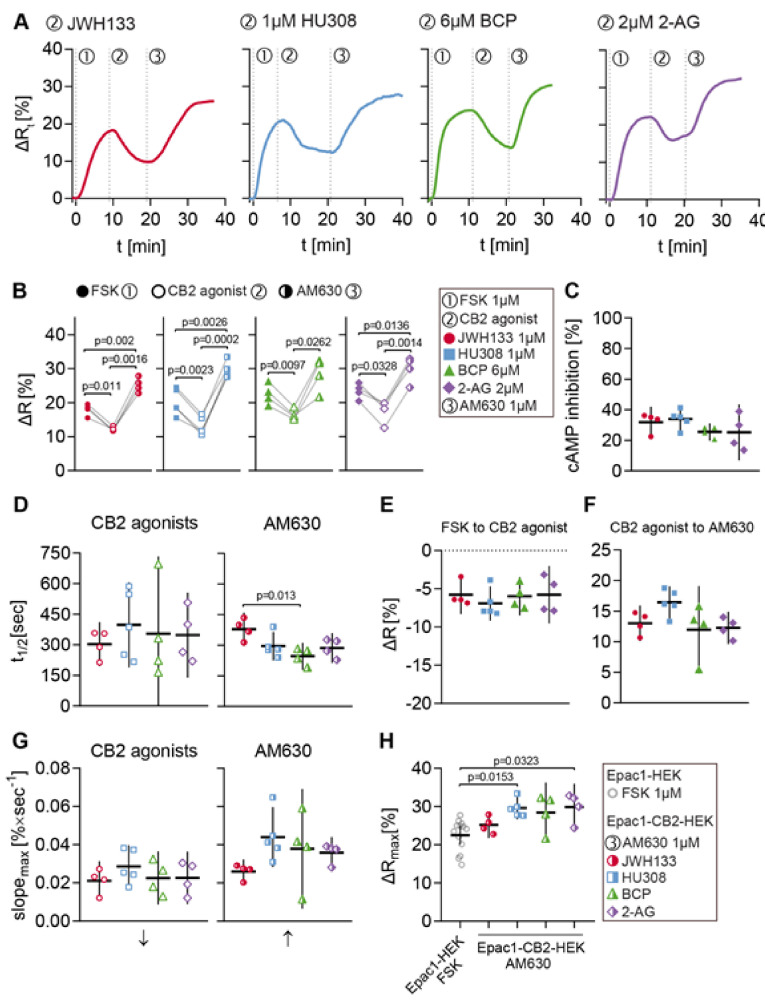
FRET responses to CB2 agonists and AM630 in Epac1-CB2-HEK type R cells. (**A**) Representative single cell FRET ratio (∆Rt) traces of Epac1-CB2-HEK cells stimulated with 1 µM FSK (1), 1 µM then JWH133 (*n* = 4), HU308 (*n* = 5), BCP (*n* = 4) or 2-AG (*n* = 4) (2), and 1 µM AM630 (3). (**B**) Baseline FRET (∆R) values after FSK (full icon), CB2 agonists (hollow icon; JWH133: red circles; HU308: blue squares; BCP: green triangles; 2-AG: violet diamonds) and AM630 (half-full icon). Data were analyzed using Two-Way ANOVA with repeated measures and Sidak-adjusted p-values. (**C**) Inhibition of FSK-elicited cAMP production by different CB2 agonists. (**D**) t1/2 values of FRET responses until CB2 agonist (left) and AM630 (right) baselines from stimulations with different CB2 agonists. (**E**) ∆R differences from FSK to CB2 agonist and CB2 agonist to AM630 (**F**) baselines from stimulations with different CB2 agonists. (G) max. slope of FRET responses (arrows indicate direction of slope) until CB2 agonist (left) and AM630 (right) baselines from stimulations with different CB2 agonists. (**H**) Comparison between max. ∆R responses from Epac1-HEK cells stimulated with 1 µM FSK (*n* = 13) and Epac1-CB2-HEK cells after the AM630 response. Data were analyzed using Nested One-Way ANOVA with Tukey (**C**–**G**) or Dunnett (**H**) adjusted *p*-values. Each data point n represents the average of all cells in one recording with at least three responding cells. Mean ± 95%CI.

The same stimulation protocol was applied in order to evaluate the generated cell model regarding its functionality and reliability of characterizing CB2-mediated cAMP signaling and CB2 ligands. A variety of CB2 agonists was used to investigate the universality of observed cellular responses to CB2 activation. The agonists used in these experiments were again the synthetic selective CB2 agonists JWH133 and HU308, the terpenoid β-caryophyllene, as well as the endocannabinoid and CB1/CB2 agonist 2-AG. Following agonist stimulation, the cells were treated with 1 µM of the selective CB2 inverse agonist/antagonist AM630 to specifically block the agonist response. The FRET data summarized in Figure 6 originate from all Epac1-CB2-HEK cell recordings with at least three individual type R cells/ROIs.

Representative ∆Rt line plots from different ROIs that were stimulated with FSK (1), JWH133, HU308, BCP or 2-AG (2), and AM630 (3) are displayed in Figure 6A. The cells showed a ∆R increase by about 20% after stimulation with 1 µM FSK and following stimulation with CB2 agonist a ∆R decrease between 5 and 10% was seen. This ∆R decrease represents the direct inhibition of FSK-elicited cAMP production via CB2 activation in living Epac1-CB2-HEK cells. Subsequent stimulation with 1 µM AM630 resulted in a ∆R increase of approximately 15%. The blockage of CB2-mediated cAMP inhibition via AM630 shows the specificity of the agonist response and the reversibility of receptor activation.

The summarized ∆R extreme values after each stimulation time point (within one recording) show the similarity between type R recordings from different CB2-agonist stimulation protocols (Figure 6B). Corresponding analysis of ∆R differences between each baseline after substance stimulation revealed similar mean values between recordings from different CB2-agonists that ranged between a decrease of 5 to 7% (JWH133: M = −5.77, 95%CI = −8.3, −3.24; HU308: M = −6.92, 95%CI = −9.17, −4.66; BCP: M = −5.98, 95%CI = −8.49, −3.47; 2-AG: M = −5.78, 95%CI = −9.53, −2.03) (Figure 6E). This was also reflected by the small variation of overall cAMP inhibition that was caused by the different CB2 agonists (JWH133: M = 31.85, 95%CI = 21.70, 41.99; HU308: M = 33.95, 95%CI = 26.41, 41.49; BCP: M = 25.50, 95%CI = 19.85, 31.15; 2-AG: M = 25.20, 95%CI = 7.02, 43.38) (Figure 6C). On average, agonist-elicited response parameters max. slope and time to half-maximum did not show significant differences between recordings (Figure 6D,G).

The FRET responses of Epac1-CB2-HEK cells to 1 µM of CB2 inverse agonist AM630 showed more variability, depending on precedent CB2 agonist stimulation (Figure 6D,F,G). The mean differences in ∆R between CB2 agonist and AM630 baseline ranged between 10 and 17% (JWH133: M = 13.00, 95%CI = 10.10, 15.91; HU308: M = 16.42, 95%CI = 13.80, 19.04; BCP: M = 11.94, 95%CI = 4.81, 19.07; 2-AG: M = 12.27, 95%CI = 9.63, 14.90) (Figure 6F). AM630 responses in JWH133 stimulated cells showed on average a larger half-time (M = 379, 95%CI = 297, 461) and lower max. slope values (M = 0.0261, 95%CI = 0.0198, 0.0323) as compared to the other agonist stimulation protocols (Figure 6D,G). The difference in AM630 response half-time between JWH133 and BCP stimulated cells was most prominent (JWH133 vs. BCP: M = 133, 95%CI = 28, 239, *p* = 0.0125) (Figure 6D).

The comparison of the maximal FRET response ∆Rmax in Epac1-HEK cells after stimulation with 1 µM FSK and the end-point ∆R values of Epac1-CB2-HEK cells after the completion of the stimulation protocol revealed that after stimulation with AM630, Epac1-CB2HEK cells had higher cAMP levels than control cells stimulated with FSK (Figure 6H). This difference in ∆R was most evident in Epac1-CB2-HEK cells from the HU308 (Epac1-HEK FSK vs. HU308: M = −5.96, 95%CI = −10.96, −0.96, *p* = 0.0153) and 2-AG (Epac1-HEK FSK vs. 2-AG: M = −6.02, 95%CI = −11.64, −0.41, *p* = 0.0323) treatment (Figure 6H). This observation was also made when comparing the ∆R values after FSK stimulation to ∆R values after AM630 stimulation within the same recording from Epac1-CB2-HEK cells (Figure 6B) and additionally hints towards the presence of constitutive CB2 activity in Epac1-CB2-HEK cells that show a type R response.

Acquired FRET data from Epac1-CB2-HEK cells that were stimulated with different CB2 agonists showed that detecting the inhibition of cAMP production in live cells via the activation of the Gαi -coupled receptor CB2 was possible with the implemented imaging pipeline. Therefore, our experimental setup using the Epac1-CB2-HEK cell model is a valid system for investigating and detecting intracellular cAMP changes in living cells upon CB2 activation or blockage that are not detectable in multicell assays.

## 3. Discussion

In the scope of this work, a HEK293 cell model with the expression of human CB2 and the FRET-based cAMP biosensor Epac1-camps was generated in order to dynamically monitor CB2 mediated cAMP signaling. With this cell model, it was possible to detect a cellular cAMP-FRET response to all applied CB2 agonist (JWH133, HU308, BCP, and 2-AG) showing high similarity in all analyzed response parameters and overall cAMP inhibition. Remarkably, none of the CB2 agonists completely inhibited all FSK-elicited cAMP production in live cells in these assays. Although there was some variation and a higher variety in cellular responses towards the CB2 inverse agonist AM630, which was applied after CB2 agonists, a clear blockage of CB2-mediated cAMP inhibition was seen in all analyzed recordings.

The measurement of cAMP levels on a single cell level revealed the variability across cells of the generated cell model. Three different types of cellular responses to the CB2 stimulation protocol were observed that possibly reflect different receptor expression levels and/or conformations. From the observation of an inhibitory effect of cAMP production that is elicited by CB2 agonists, it can be deduced that type R responder cells have CB2 receptors in an equilibrium state that allows for the agonists to shift it towards more receptor activity visible in the Gαi -mediated inhibition of ACs.

Cells that only responded to the CB2 inverse agonist AM630 (type CA) supposedly have a different composition of receptor conformations with a high degree of constitutively active receptors. AM630 as a CB2 protean ligand binds and stabilizes inactive receptor conformations with high affinity [12] and, therefore, shifts the activity equilibrium towards inactivity. In a system with high basal activity, corroborated by the inability of CB2 agonists to elicit additional activity, this will lead to pronounced inverse agonism, as seen in type CA cells [31].

The presence of constitutively active receptor forms is also seen in type R cells as AM630 stimulation led to higher intracellular cAMP levels when compared to the control cells. In CB2 cell models with heterologous expression, a high degree of constitutive activity has been observed before [8,11,12] and, therefore, might be a characteristic of the receptor.

The faster response to 1 µM FSK in all Epac1-CB2-HEK cell response types as compared to Epac1-HEK cells might indicate that heterologous CB2 expression alters the available G-protein pool. It has been reported that free Gαi subunits can cause effects at ACs that are independent of receptor activation [32]. Through the presence of, e.g., spontaneously inactive CB2 that couple Gαi subunits [33], but do not elicit a signaling response, the pool of free Gαi might be reduced, leading to a faster cAMP production after direct AC activation with FSK in Epac1-CB2-HEK cells. However, this would also mean that type N responders, which do not show a response to either CB2 agonist or AM630, express CB2, because the faster and stronger cAMP production after FSK as compared to Epac1-HEK cells was also seen in these response types. A possible explanation for this observation could be the sensitivity of Epac1-camps that might be too low to detect small changes in cAMP produced by a small amount of active CB2 receptors. A more sensitive Epac-based FRET-sensor, like, e.g., mTq2-Epac-c p mVc p mV (H187) [29,34], could elucidate whether there is, in fact, a very small CB2-agonist induced inhibition of cAMP or not. Other than that, the FSK responses did not seem to differ significantly between Epac1-CB2-HEK cell response types and Epac1-HEK cells.

All of the CB2 agonists applied caused an inhibition of around 30% of cAMP produced by 1 µM FSK. This response was further blocked by CB2 inverse agonist/antagonist AM630, indicating the CB2 specificity of the observed cAMP inhibition. The similar maximum effect of all agonists, including the synthetic cannabinoids JWH133 and HU308 that are recommended to use as CB2 selective reference agonists [13], suggests that the observed response is the maximum of CB2-mediated Gαi inhibition of ACs in Epac1-CB2-HEK cells. In this study, BCP was confirmed as a functional CB2 agonist [17,18], which elicited the same effect on intracellular cAMP levels in live Epac1-CB2-HEK cells, like the endogenous CB2 ligand 2-AG.

Previous studies of CB2-mediated cAMP signaling have exclusively used multicell approaches to determine the inhibition of cAMP production after CB2 activation as either endpoint [13,21,35] or as live measures [19,23]. Similar to this study, FSK and, often, also the PDE inhibitor IBMX (3-isobutyl-1-methylxanthine), are added to the stimulation with the receptor ligands to increase basal cAMP levels. A study that also uses 1 µM FSK as pre-stimulant and IBMX was additionally published by Dhopeshwarkar and Mackie [21]. They showed around 60% inhibition of FSK-elicited cAMP production after five minutes stimulation time by JWH133 (61 ± 1.1%, mean ± SEM), HU308 (60 ± 3.4%) and 39 ± 0.7% for 2-AG, respectively. However, these authors observed a relatively low BCP effect of 5 ± 1.1% in contrast to our 25 ± 1.7% (mean±SEM) cAMP inhibition. Recent studies using AtT20-CB2 cells even reported an absence of BCP effects on signaling pathways involving potassium channel dependent hyperpolarization measured by FLIPR^®^ membrane potential dye [20] and on the modification of cAMP signaling [19]. The latter study used a HA-3TCS-hCB2 HEK cell line with transient transfections of the CAMYEL biosensor, which is a BRET dependent cAMP sensor with YFP-Epac-RLuc [19]. In contrast to our experimental set-up using live imaging on single cells, the other studies were performed on cell populations. Possibly our results of variability across cells are also valid for other cell lines. If this would be the case and if a cell model has variations in the percentage of responder cells in relation to non-responder and constitutive active cells, they will not be detected by multicell assays and they could result in different total ligand effects.

Different CB2 agonists might have varying dynamics and signaling bias that is dependent on CB2 phosphorylation that could originate from PKA activation downstream of cAMP production by FSK [29,30,31]. Because the highly potent and cAMP biased CB1/CB2 agonist CP55,940 is regularly used as a reference ligand to establish experimental protocols [13,21], with regard to this, previously reported agonist differences have to be interpreted accordingly.

With endpoint measurements, it is unclear whether the cAMP concentration at a given timepoint has already reached a stable baseline or a transient extreme. Through the live imaging of cAMP levels in this present work, it was possible to stimulate the cells once they reached a new baseline after FSK or CB2 agonist application and, therefore, detect effects on net intracellular cAMP concentration that are attributable to the stimulants.

Although monitoring the live dynamics of cAMP allows for the improved dissection of CB2 responses to stimulants, the effects of the FSK stimulation can still overlap and influence the interpretation of FRET responses to CB2 agonists. The reported low levels of intracellular cAMP in HEK293 cells [36,37] and the sensitivity of the Epac1-camps FRET sensor [26,29] required the use of FSK to elevate basal cAMP levels in order to detect its inhibition. Through this, the imaging time period had to be increased for measurements on Epac1-CB2-HEK cells, which possibly led to further variability.

Taken together, we have managed to visualize and quantify the cAMP responses of the Gαi-coupled CB2 receptor while using a live FRET imaging technique on single cells level in the heterologous cell system.

Future studies using this method will clarify whether the experimental set-up will enable live cell FRET imaging of cAMP responses in endogenous CB2 expressing cells.

## 4. Materials and Methods

### 4.1. Cells, Reagents and Software

Cells: HEK293 by CLS cell line service GmbH, Eppelheim, Germany, Cat# 300192/p777_HEK293; Epac1-camps HEK293 (V. Nikolaev).

Chemicals: n-dodecyl β-d-maltoside (DDM) #D4641 (Sigma–Aldrich, Steinheim, Germany), Forskolin (FSK) #F6886 (Sigma–Aldrich, Steinheim, Germany), poly-L-Lysine (PLL) #P6282 (Sigma–Aldrich, Steinheim, Germany).

Cell Culture Reagents: DMEM #41966-029 (Life Technologies, Paisley, UK), HBSS #H6648, (Sigma–Aldrich, Steinheim, Germany), Opti-MEM #31985-062 (Life Technologies, New York, Grand Island, NY, USA), Lipofectamine^®^2000 #11668-019 (Life Technologies, California, Carlsbad, CA, USA), Penicillin-Streptomycin #P4333 (Sigma–Aldrich, Steinheim, Germany), Hygromycin B #CP12.2 (Roth), G-418 #4727878001 (Roche, Mannheim, Germany), Fetal Bovine Serum #P30-3312 (Pan Biotech, Aidenbach, Germany), Trypsin #T7409 (Sigma–Aldrich, Steinheim, Germany).

Cannabinoids: 2-AG #62160 (Cayman, Michigan, Ann Arbor, MI, USA), beta-caryophyllene #C9653 (Sigma–Aldrich, Steinheim, Germany), AM630 #1120 (Tocris, Bristol, UK), JWH-133 #1343 (Tocris, Bristol, UK), HU-308 #3088 (Tocris, Bristol, UK).

Antibodies: Rabbit anti-FLAG # F7425 (Sigma–Aldrich, Steinheim, Germany), rabbit anti-actin # A2066 (Sigma–Aldrich, Steinheim, Germany), donkey anti-rabbit IRDye^®^ 680LT #926-68023 (LI-COR Biosciences, Nebraska, Lincoln, NE, USA), donkey anti-rabbit IRDye^®^ 800CW #926-32213 (LI-COR Biosciences, Nebraska, Lincoln, NE, USA), mouse anti-FLAG^®^ M2 # F1804 (Sigma–Aldrich, Steinheim, Germany), anti-mouse IgG, Alexa Fluor^®^ 488 and 594 (Thermo Fisher Scientific, Oregon, Eugene, OR, USA).

Software: ImageJ [38,39] ver. 1.43 and 1.51; ImageJ Plugin FRET analysis [40] ImageJ Plugin MicroManager (ver. 1.4.5); ImageJ Plugin MultiStackReg ver. 1.45; R (The R Foundation, ver. 3.5.1); R Studio (RStudio, Inc., ver. 1.1.383, Richmond Hill, ON, L4C 3C7, CANADA); Prism (GraphPad Software, ver. 8.0.2, San Diego, CA 92108, USA).

### 4.2. HEK293 Cell Culture and Preparation for Downstream Assays

Epac1-HEK cells were maintained at 37 ºC and 5% CO_2_ and kept in DMEM supplemented with 10% FBS and 1% penicillin/streptomycin (P/S). HEK293 cells stably expressing FRET-cAMP biosensor Epac1-camps were kept in the described HEK293-medium with additional 400 µg/µL hygromycin B. For double stably transfected HEK293 with Epac1-camps and FLAG-hCB2, DMEM plus 10% FBS and 1% P/S was additionally supplemented with 400 µg/µL hygromycin B and 600 µg/µL G418. The preparation of cell lysates for downstream assays such as Western Blot was done by removing the medium and applying ice-cold n-dodecyl β-D-maltoside (DDM) lysis buffer directly onto the cells. The preparation of extracts was described in detail before [41]. The lysates were then transferred into reaction tubes, homogenized with a 27G syringe, incubated for 30 min. at 4 ºC on a rotor and centrifuged for 15 min. at 13,000 rpm and 4 ºC. The supernatant was used immediately for further assays or stored at −20 °C.

### 4.3. SDS-Polyacrylamide Gel Electrophoresis (SDS-PAGE) and Immunoblotting

Protein extract separation was performed by 10% SDS-PAGE and proteins were then transferred onto a nitrocellulose membrane at 95 V for 2 h at 4 °C. After the successful transfer of proteins, indicated by a visible protein ladder, the membrane was blocked in Western blot blocking solution for 1 h at room temperature on a shaker. Following blocking, the membranes were incubated over night at 4 °C on a shaker in primary antibody solution (Rabbit anti-FLAG Sigma–Aldrich Cat# F7425 1:1000 in TBST and rabbit anti-actin Sigma–Aldrich Cat# A2066 1:1000 in TBST). Subsequently, membranes were washed three times with TBST for 5 min. at room temperature on a shaker and then incubated with either IRDye^®^ 680LT or IRDye^®^ 800CW-coupled secondary antibodies in TBST (1:10,000) for 1 h at room temperature on a shaker. Membranes were then washed with TBST. The detection of target protein bands from the wet membrane was done using the Odyssey^®^ CLx imaging system and band intensities were quantified using Image Studio Lite.

### 4.4. Immunocytochemistry 

The cells on coverslips were washed with PBS and then fixed with 4% PFA for 20 min. at room temperature. HEK293 transfected with FLAG-hCB2, Epac1 and Epac1-CB2-HEK cells were then blocked with 5% normal goat serum in PBS for 1 h. Primary antibodies used for all HEK293 cell model systems were rabbit anti-CB2 (1:300), mouse anti-HA (1:300) and rabbit anti-FLAG (1:300). Fixed cells were incubated with primary antibody for 24 h at 4 °C on a shaker and then washed three times for 5 min. with cold DPBS. Secondary antibody solutions were prepared with anti-mouse IgG, Alexa Fluor^®^ 488 and 594 and anti-rabbit IgG, Alexa Fluor^®^ 488 and 594 in DPBS, and cells were incubated for 2 h at room temperature on a shaker in the dark. Coverslips were then washed three times for 15 min. with DPBS and mounted onto glass slides with ProLongTM Gold Antifade Mountant medium (Sigma–Aldrich) and dried at room temperature overnight in the dark. Slides were then stored at 4 °C until imaging on a confocal microscope.

### 4.5. Live Cell FRET Imaging

Live cell FRET imaging was carried out as previously described [37,40,42]. Epac1 and Epac1-CB2-HEK cells were plated on poly -L-Lysin -coated 22 mm glass cover slips in six-well-plates at a density of 0.1 × 10^6^ cells per well and then incubated for 48 h. On the imaging day, coverslips with Epac1-, Epac1-CB2-HEK cells were transferred into a cell imaging chamber, washed one time using FRET imaging buffer, and then kept in a minimum of 400 µL FRET imaging buffer at room temperature during imaging. The imaging chamber was secured on the stage of an inverted microscope equipped with a 63× oil objective. The cells were excited using a 440 nm LED and fluorescent emission was detected with a dualemission photometry system using a CCD camera. The excitation time and image acquisition were controlled and synchronized while using the Micro-Manager plugin for ImageJ [38]. Images were acquired every 20 s and 50 ms to 100 ms excitation time, depending on the expression of Epac1-camps. Live fluorescence intensities and the raw FRET ratio were monitored during the recording and the stimulants were applied after a baseline was reached. Cell stimulants (dissolved in DMSO and further diluted in FRET imaging buffer) were then carefully added to the cell imaging chamber in appropriate concentrations (maximum concentration of DMSO on cells: 0.05%).

### 4.6. FRET Data Analysis

The acquired time lapse emission channel images were processed while using ImageJ and following calculations and FRET response analyzes were done using self-made scripts in R. For image noise reduction a Kalman stack filter was applied and using the MultiStackReg plugin (rigid body transformation) the two channels were aligned. ROIs on cells were drawn where Epac1-camps expression was homogenous and movement was minimal throughout the recording. The integrated density (IntDen), area and average grey (AveGrey) value of each ROI for each frame were measured, and the total corrected cellular fluorescence (TCCF) was calculated according to [43]:

TCCF = ROI IntDen − ROI Area × BG AveGrey(1)

After the individual channels were inspected for excessive photobleaching and correct opposing fluorescence intensity changes, the FRET ratio R was calculated, as follows:

R = TCCF YFP/TCCF CFP − B(2)

Where B is the bleedthrough correction factor of CFP emission into the YFP channel determined for the imaging setup. For statistical analysis and data visualization, the FRET ratio for each ROI over time was normalized to its pre-stimulation baseline R0, subtracted from 1, and then multiplied by 100 in order to depict the decrease in FRET upon increased intracellular cAMP levels as a percentage increase or vice versa. It was calculated, as follows:

∆Rt [%] = (1 − Rt / R0) × 100(3)

For each FRET response after application of a specific stimulant, local maxima and minima were detected and, for each of these response curves, the maximum slope as well as the time to 50% of the signal’s amplitude were determined. The max. slope values are always depicted without a sign. Plots featuring max. slope values are supplied with information on the direction of the response.

For CB2 activation experiments in Epac1-CB2-HEK cells, ROIs/cells showing at least 10% cAMP inhibition after CB2 agonist application and a blockage of the inhibition via AM630 (max. slope of response: ≥0.01) are considered to be responders (R). Cells with less than 10% cAMP inhibition after agonist stimulation, but a clear response to AM630. show a response pattern of high constitutive activity (CA) of CB2 receptors. If a cell showed no response to either CB2 agonist or inverse agonist/antagonist with a linear increase of ∆Rt after reaching the FSK baseline comparable to Epac1-HEK cells, the cell is categorized as a non-responder (N). In order to avoid the pitfalls of pseudoreplication in recordings with multiple cells from Epac1-HEK and Epac1-CB2-HEK cells, the FRET response parameters of a minimum of three cells with the same response pattern in one recording were averaged. The inhibition of FSK-mediated cyclic AMP production by CB2 agonists was calculated, as follows:

c AMP inhibition [%] = (∆R Agonist / ∆R FSK − 1) × 100(4)

### 4.7. Statistical Analysis

All of the statistical analyses were carried out using GraphPad Prism and applied statistical test in order to estimate differences between sample groups are given with the result figures. The Shapiro–Wilk test was used to determine the normality of a given sample population. Epac1-HEK and Epac1-CB2-HEK cell FRET data were analyzed using Nested-models with ROIs/cells nested within recordings.

## 5. Conclusions

In the scope of this work, a cell model was generated that allowed for the live cell FRET imaging of CB2-mediated cAMP dynamics while using the cAMP biosensor Epac1-camps. This model proved to be valuable in the detection of CB2 agonist-elicited inhibition of cAMP production via Gαi subunits and their blockage with a CB2 inverse agonist. The cell’s dynamic response to the stimuli provides insights on the nature of heterologous CB2 expression systems that most likely show constitutive activity, and contributes to the understanding of Gαi -mediated GPCR signaling in living cells. Future research should try to implement FRET imaging for primary cells that endogenously express CB2 and they are potentially important for CB2 function in vivo, in order to study CB2-mediated cAMP signaling in a physiological setting.
